# Diversity of Coronaviruses in Wild Representatives of the *Aves* Class in Poland

**DOI:** 10.3390/v13081497

**Published:** 2021-07-29

**Authors:** Katarzyna Domańska-Blicharz, Justyna Miłek-Krupa, Anna Pikuła

**Affiliations:** Department of Poultry Diseases, National Veterinary Research Institute, Al. Partyzantów 57, 24-100 Puławy, Poland; mjustyna6@gmail.com (J.M.-K.); anna.pikula@piwet.pulawy.pl (A.P.)

**Keywords:** wild birds, coronavirus, Poland

## Abstract

The revealed prevalence of coronaviruses in wild bird populations in Poland was 4.15% and the main reservoirs were birds from orders *Anseriformes* and *Charadriiformes*, with a prevalence of 3.51% and 5.59%, respectively. Gammacoronaviruses were detected more often than deltacoronaviruses, with detection rates of 3.5% and 0.7%, respectively. Gammacoronaviruses were detected in birds belonging to six orders, including *Anseriformes*, *Charadriiformes*, *Columbiformes*, *Galliformes*, *Gruiformes*, and *Passeriformes*, indicating a relatively wide host range. Interestingly, this was the only coronavirus detected in *Anseriformes* (3.51%), while in *Charadriiformes*, the prevalence was 3.1%. The identified gammacoronaviruses belonged to the *Igacovirus* and *Brangacovirus* subgeneras. Most of these were igacoviruses and formed a common phylogenetic group with a Duck Coronavirus 2714 and two with an Avian Coronavirus/Avian Coronavirus9203, while the viruses from the pigeons formed a distinct “pigeon-like” group, not yet officially represented. The presence of deltacoronaviruses was detected in birds belonging to three orders, *Charadriiformes*, *Galliformes*, and *Suliformes* indicating a narrower host range. Most identified deltacoronaviruses belonged to the *Buldecovirus* subgenus, while only one belonged to *Herdecovirus*. Interestingly, the majority of buldecoviruses were identified in gulls, and they formed a distinct phylogenetic lineage not represented by any officially ratified virus species. Another separate group of buldecoviruses, also not represented by the official species, was formed by a virus identified in a common snipe. Only one identified buldecovirus (from common pheasant) formed a group with the ratified species Coronavirus HKU15. The results obtained indicate the high diversity of detected coronaviruses, and thus also the need to update their taxonomy (establishing new representative virus species). The serological studies performed revealed antibodies against an infectious bronchitis virus in the sera of white storks and mallards.

## 1. Introduction

Viruses of the *Orthocoronavirinae* subfamily in the *Coronaviridae* family and the *Nidovirales* order have an envelope equipped with protruding structures on their surface called spikes and a positive-sense single-strand RNA genome of approximately 27–32 kb in size. The genomes of all coronaviruses have a similar structure and organization, but also display unique genus or even strain-specific genomic structures including accessory genes. Generally, the 5′-proximal two-thirds of the genome encode a set of proteins responsible for viral RNA replication and transcription, including RNA-dependent RNA polymerase (RdRp). At the 3′ end are genes that, among other products, encode four major structural proteins, namely: spike (S), envelope (E), matrix (M), and nucleocapsid (N) [1]. The *Orthocoronavirinae* subfamily incudes viruses from four genera, namely *Alpha-*, *Beta-*, *Gamma-* and *Delta-coronavirus*. The basis of the classification of coronaviruses is the analysis of the five most conserved replicative proteins encoded by open reading frames 1a and 1b (ORF1a/1b): nsp5-3CLpro, nsp12-NiRAN, nsp12-RdRp, nsp13-ZBD, and nsp13-HEL1. Only gammacoronaviruses (gammaCoVs) and deltacoronaviruses (deltaCoVs) infect bird species, but some of them can also infect mammals [2]. According to the Virus Taxonomy: 2020 Release, two of the existing three *Gammacoronavirus* subgenera, namely *Igacovirus* and *Brangacovirus*, were identified in birds, the third, *Cegacovirus*, was identified in a marine mammal, the beluga whale (SW1 virus) [3]. Three species represent the *Igacovirus* subgenus: avian coronavirus (AvCoV) and avian coronavirus 9203 (AvCoV9203), which together comprise all the genotypes of infectious bronchitis virus (IBV), turkey and guinea fowl coronaviruses, and the third species duck coronavirus 2714 (DuCoV2714) firstly described in healthy breeding ducks in China in 2013 [4,5,6]. The only species of the subgenus *Brangacovirus*, the geese coronavirus CB17 (BcanCoVCB17), was identified in cloacal swabs taken from Canadian geese found in the polar region of Cambridge Bay, Canada, where mass die-offs were observed in the fall of 2017 [7]. The youngest in the coronavirus taxonomy, the *Deltacoronavirus* genus, was established in 2011, and its subdivision into lower taxonomic groups has changed over the past 10 years. Currently, three subgenera have been determined—*Andecovirus*, *Buldecovirus*, and *Herdecovirus*. Two subgenera, *Andecovirus* and *Herdecovirus*, are represented by single species of viruses, wigeon coronavirus HKU20 (WeCoV HKU20) and night heron coronavirus HKU19 (NhCoV HKU19), respectively. The *Buldecovirus* genus is the most diverse, which includes five different species: bulbul coronavirus HKU11 (BuCoV HKU11), common moorhen coronavirus HKU21 (CMCoV HKU21), coronavirus HKU15 (CoV HKU15), munia coronavirus HKU13 (MuCoV HKU13), and white-eye coronavirus HKU16 (WeCoV HKU16). All of these deltacoronaviral strains were identified in the samples from animals collected between 2007 and 2011 in various locations of Hong Kong Special Administrative Region [8,9]. An interesting case is the only representative case of deltacoronaviruses in mammals, CoV HKU15. It was first identified in 2009 in pig fecal samples in Asia, but its role in the etiology of diarrhea was only confirmed in 2014, and it is now known to occur worldwide [9,10]. Interestingly, viruses similar to porcine deltacoronaviruses have been found in sparrows and quails and belong to the same species CoV HKU15 (with amino acid homology in the domains used for species separation >96%), which may indicate that transmission from birds to mammals happened relatively recently [11].

Coronaviruses have been identified in 15 orders of *Aves*, comprising 30 families and 108 species of wild birds. They are most commonly found in waterfowl and shorebirds, especially in *Charadriiformes* and *Anseriformes*, which are considered as a reservoir of avian influenza viruses (AIV) [12]. However, this observation could be because the same samples were usually tested (i.e., coronaviruses in samples originally collected for the avian influenza survey). The prevalence of coronaviruses in wild birds varies significantly from 0.3% to 50%, mainly depending on the detection methods used, but also on the season and location of the sampling and probably also on other factors such as the age or taxonomic group of the birds, as well as their behavior (migratory versus resident, waterfowl, and other).

In previous studies, we described the presence of gammaCoV in 3.5% of wild birds of the *Anseriformes*, *Charadriiformes*, and *Galliformes* orders sampled in Poland [13]. The detection tools used at that time targeted different fragments of the IBV genome, 3′UTR fragment as screening method and gene 3, viral replicase (RdRp), and N genes used for molecular characterization of the detected viruses. This is why the viruses detected were referred to as IBV-like, and their genes were highly similar to the most frequently detected lineages of IBV in this geographical region, i.e., Mass, 793/B, and QX. This paper presents the results from the molecular survey and phylogenetic analysis of the coronaviruses detected in wild birds sampled in Poland, with the diagnostic method used aimed at detecting all of the genera of coronaviruses.

## 2. Materials and Methods

### 2.1. Ethics Statements

The bird samples were collected by ornithologists, with appropriate licenses and permits given by relevant agencies of the provisional and central government. All of the actions taken during this study were carried out in accordance with the recommendations contained in the guidelines of the World Organization for Animal Health [14].

### 2.2. Sample Collection

Cloacal/fecal swabs collected individually from 3998 birds between 2011 and 2018, and additionally 400 pooled swabs (up to five birds of the same species collected at the same time and location) gathered in an earlier period between 2009 and 2011 were used for the study. These samples were previously tested for AIV within active surveillance in Poland, and were mainly from live water and shorebirds, representing 80 species belonging to 15 orders (Table 1). More than 91% of the samples were tested from individuals, the rest were used as pooled samples. The swabs from birds of *Anseriformes* constituted 70.6% of all individual samples, of which 39.2% were from mallards (*n* = 1585) and 28.0% from mute swans (*n* = 1119). In turn, 25.5% of the samples originated from the *Charadriiformes*, the most numerous of which were the black-headed gulls (19.3%, *n* = 773) and common and herring gulls (3.6%, *n* = 142 and 2.1%, *n* = 82, respectively). The remaining 13 orders were represented by much smaller number of samples, with the exception of *Columbiformes* and *Ciconiiformes*, the samples of which constituted about 0.5% of the total. The structure of the tested pooled samples was similar. During the three-year period, the samples collected from *Anseriformes* and *Charadriiformes* constituted 89.5% of all of those tested in this category. Of the 72.8% of the samples from *Anseriformes*, 25.0% (*n* = 100) were from graylag geese, 21.3% (*n* = 85) from mute swans, 16.3% (*n* = 65) from mallards, and 6.8% (*n* = 27) from bean geese. Among the 16.8% of samples from *Charadriiformes*, the most numerous were black-headed (7.5%, *n* = 30) and common gulls (6.3%, *n* = 25). The samples were collected using dry swabs or swabs wet with a viral transport medium (Copan UTM, Brescia, Italy). After collection, the samples were immediately placed in field refrigerators and then transported to the laboratory at the National Veterinary Research Institute under cooling conditions.

In addition, the serum samples collected in the frame of serological AIV monitoring collected for three years between 2011 and 2013 from 264 birds, including 87 mallards, 88 white storks, and 89 mute swans were used in the study.

### 2.3. Molecular Detection of CoVs

Dry swabs were immersed in 2 mL of phosphate-buffered saline (Biomed, Lublin, Poland) and incubated for 1–2 h at room temperature. All of the swab fluids were clarified by centrifugation (15 min at 3000× *g*) and before the process of RNA isolation were pooled (up to five birds of the same species collected at the same time and location). In the case of a positive result, the samples were re-isolated individually as described below. The RNA from 200 µL of obtained fluid was extracted into a volume of 20 µL using an RNeasy Mini Kit (Qiagen, Hilden, Germany) or Viral Mini Kit (Syngen, Wroclaw, Poland), according to the manufacturers’ protocols. The samples were tested for CoV presence with an RT-PCR assay in a nested format using degenerate primers [15]. The assay amplified a 555-nucleotide fragment of the viral replicase gene of coronaviruses found in humans and other mammals such as bats or pigs, but also in birds. The PCRs were performed in a final volume of 25 µL. In the first step of the nested PCR, the One-Step RT-PCR kit (Qiagen, Hilden, Germany) was used. In the second step, the Platinum™ Taq DNA Polymerase kit (Invitrogen, Carlsbad, CA, USA) was used with a volume of 2.5 µL of 1:5 (*v*/*v*) dilution of PCR product. The vaccine IBV 4/91 strain (Nobilis^®^ IB 4/91, Intervet International B.V., Boxmeer, The Netherlands) served as a positive control. The PCR-positive samples were identified by agarose gel electrophoresis and then sequenced using 3500 Genetic Analyzer (Applied Biosystems, Foster City, CA, USA). If multiple bands were visualized, amplicons of the appropriate size were excised from the gel and purified with the QIA quick gel extraction kit (Qiagen, Hilden, Germany) and subsequently sequenced.

### 2.4. Sequence and Phylogenetic Analysis

The obtained forward and reverse sequences were trimmed and assembled into consensus using Geneious v11.1.3 (Biomatters, Ltd., Auckland, New Zealand). Only sequences of a good quality were included for the phylogenetic analysis—those poorly aligned were eliminated. Next, the obtained sequences were analyzed in the GenBank database using the BLAST algorithm with the default parameters, and the sequences with the highest homology were downloaded for further analysis. These sequences together with 12 sequences of reference strains, including five representing individual species from three subgenera (*Igacovirus*, *Brangacovirus*, and *Cegacovirus*) of the *Gammacoronavirus* genus and seven representing individual species from three subgenera (*Andecovirus*, *Buldecovirus*, and *Herdecovirus*) of the *Deltacoronavirus* genus were then aligned using the MAFFT method in Geneious, and the alignments were then exported to MEGA software v7.0.26 [16]. A maximum likelihood (ML) phylogenetic analysis was conducted using the best-fitting nucleotide substitution models. A bootstrap test including 1000 replicates was performed for each resultant tree. The phylogenetic tree accuracy was assessed with approximate likelihood-based measures of the branch supports (Shimodaira–Hasegawa (SH) approximate likelihood ratio test (aLRT)) available in the PhyML software (http://www.atgc-montpellier.fr/phyml/; accessed on 17 November 2020).

### 2.5. Accession Numbers

The viral sequences obtained in this study have been submitted to GenBank under accession numbers MK617357-MK617517. However, in order to make the phylogenetic tree more readable, 72 virus sequences were selected from a total of 161 submitted sequences, the choice being mainly based on their representativeness among given bird order/species.

### 2.6. Serological Studies

The collected bird sera were tested using the commercial ELISA ID Screen Infectious Bronchitis Competition (IDvet, Grabels, France) according to the manufacturer’s instructions. It is the only commercial ELISA test capable of determining the antibodies of two classes, IgG and IgM, against the N protein of the most known gammacoronavirus (IBV) in the serum or eggs of chickens as well as wild birds.

### 2.7. Statistical Analyses

Statistical analyses were performed in Statistica (v.10) software (StatSoft, Cracow, Poland). The eight-year CoV prevalence was estimated with a 95% confidence interval (CI) for all of the birds and was done separately for mallards and mute swans. To assess the differences in the prevalence, a chi-square (χ^2^) test was used. A *p* < 0.05 was considered statistically significant. The proportion of CoV-positive birds versus predictor “season” grouped in periods August–November (late summer and autumn migration), December–February (wintering period), and March–July (spring migration and early summer), was also assessed for all birds and separately for mallards, mute swans, and gulls (treated as the *Laridae* subfamily).

## 3. Results

### 3.1. Molecular Surveillance of CoVs in Wild Birds

Among the 3998 individually collected samples from birds, coronavirus RNA was found in 166, which constituted 4.2% of all of the samples tested (Table 1). CoV was found in 10 bird species representing the following four orders: mallard, common teal, tufted duck, and mute swan from *Anseriformes*; black-headed, herring and common gulls, and common snipe from *Charadriiformes*; pheasant from *Galliformes*; and pigeon from *Columbiformes*. The highest number of positive results was obtained in the samples from *Anseriformes*, which accounted for 2.5% of all of those tested (*n* = 99/3998). Among these, detection rates in common teal were 11.5% (*n* = 12/104), 4.9% in mallard (*n* = 63/1566), and 2.1% in mute swan (*n* = 23/1119). The samples of *Charadriiformes* constituted slightly less, 1.4% of the positive (*n* = 57/3998) with detection rates 12.5% in common gull (*n* = 18/142), 4.5% in black-headed gull (*n* = 35/773), 3.7% in herring gull (*n* = 3/82), and as much as 20.0% in common snipe (*n* = 1/5). Coronavirus was also detected in nine pigeons, and they accounted for 0.2% of all tested. The above results indicate that the prevalence of coronaviruses in the wild bird population in Poland over the 8-year period was 4.2% (95% Cl; 3.58–4.82%). The prevalence in *Charadriiformes* was 5.6% (95% Cl; 4.34–7.17%) and was higher than in *Anseriformes*, where the value was 3.5% (95% Cl; 2.89–4.25%), and the calculated difference was statistically significant (χ^2^ = 8.32, *p* = 0.0039). Similarly, if we analyze the prevalence within *Anseriformes*, it is 4.0% (95% Cl; 3.16–5.11%) in mallards and 2.0% (95% Cl; 1.37–3.07%) in mute swans, and the calculated difference is also statistically significant (χ^2^ = 8.14, *p* = 0.0043). The number of samples from other birds is too small to make statistical calculations.

Among the 166 CoVs detected, only gamma- and delta-coronaviruses were identified. However, the number of gammaCoVs was much higher (*n* = 140/166), constituting 84.3% of all of those detected. DeltaCoVs were found in 15.7% of the positive samples (*n* = 26/166). Interestingly, all coronaviruses detected in *Anseriformes* belonged to the *Gammacoronavirus*. In contrast, different results were obtained in *Chradriiformes*, where gammaCoVs were identified in 56.1% positives (*n* = 32/57) and deltaCoVs in 43.9% (*n* = 25/57). GammaCoVs were found in black-headed gulls (*n* = 24) and common gulls (*n* = 8), while deltaCoVs were detected in black-headed gulls (*n* = 11), common gulls (*n* = 10), herring gulls (*n* = 3), and in one common snipe. These results indicate that the prevalence of gammaCoVs was 3.5% (95% Cl; 2.98–4.12%), while deltaCoVs was 0.7% (95% Cl; 0.44–0.95%), and the calculated difference was statistically significant (χ^2^ = 79.94, *p* < 0.00001). The prevalence of gammaCoVs in *Anseriformes* was 3.5% (95% Cl; 2.89–4.25%) and was only slightly higher than in *Charadriiformes*, where the value was 3.1% (95% Cl; 2.23–4.40%). In turn, the prevalence of deltaCoVs in *Charadriiformes* was 2.5% (95% Cl; 1.6–3.59%).

In turn, out of 400 pooled samples, the RNA of the coronaviruses was detected in 30 samples, which constituted 7.5% of all of those tested (Table 1). CoVs were found in 12 bird species representing the following six orders: mallard, common teal, mute swan, graylag, and bean goose from *Anseriformes*; herring and common gull, and common tern from *Charadriiformes*; and pheasant, Eurasian coot, great cormorant, and common starling from *Galliformes*, *Gruiformes*, *Suliformes*, and *Passeriformes*, respectively. In this group of samples, the most positives were among *Anseriformes* and *Charadriiformes*, accounting for 5.3% and 1.3% of all of those tested, respectively. It should be noted that these data confirm the trend shown by the individual samples. Out of the 30 CoVs detected in the pooled samples, similarly as before, gammaCoVs predominated as they were identified in 93.3% (*n* = 28/30) samples. In turn, deltaCoVs were found only in two samples (6.7%). GammaCoV was found in 21 pooled samples collected from *Anseriformes*: greylag goose (*n* = 7), mute swan (*n* = 6), mallard (*n* = 5), bean goose (*n* = 2), and common teal (*n* = 1), in four pooled samples collected from *Charadriiformes*: common gull (*n* = 3), herring gull (*n* = 1), and one sample each from *Galliformes*, *Gruiformes*, and *Passeriformes*. On the other hand, deltacoronaviruses were found in one pooled sample collected from common terns (*Charadriiformes*) and from great cormorants (*Suliformes*).

### 3.2. Phylogenetic Analysis of GammaCoV

**Figure 1:**
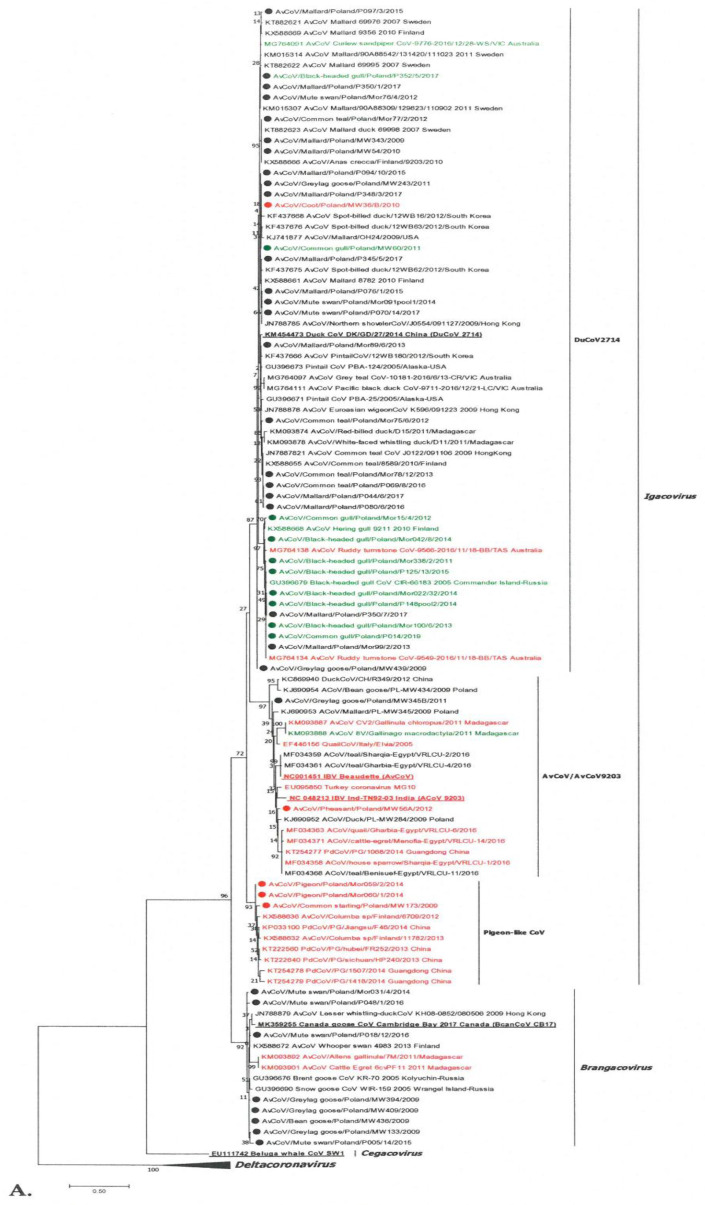

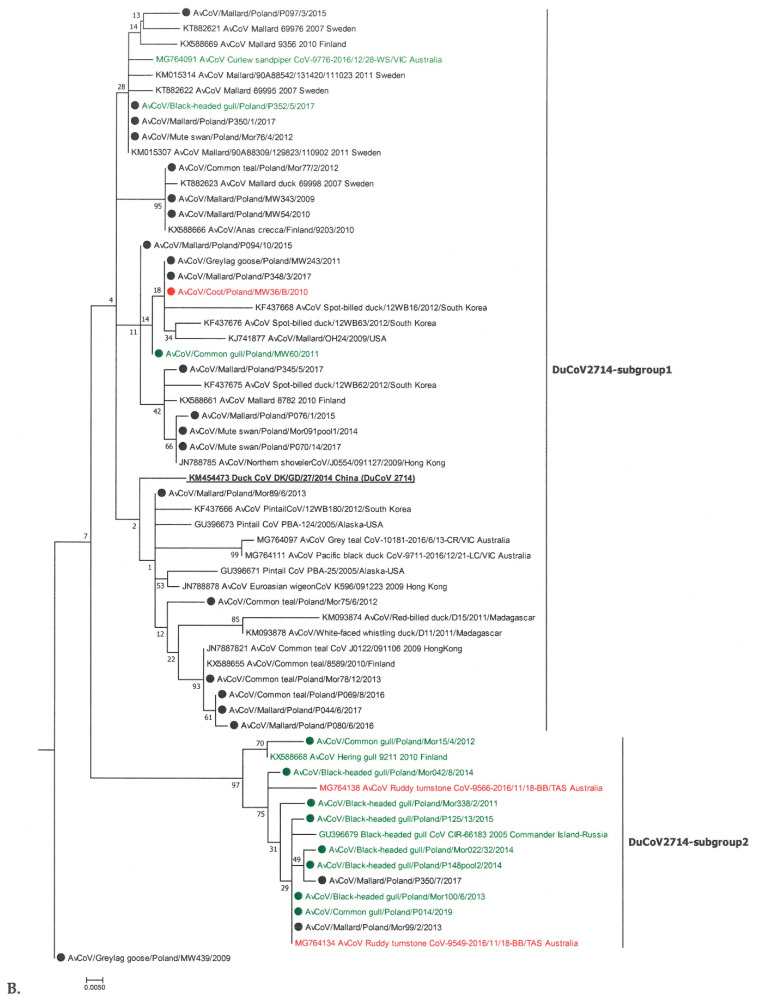
Phylogenetic analysis of gammacoronaviruses based on the replicase gene fragment. (**A**) The tree constructed for 108 gammacoronaviruses: 46 strains identified in wild birds in Poland (marked with a dot) and 62 from GenBank, including five reference strains (written in bold and underlined) representing ratified species of three subgenera (*Brangacovirus*, *Igacovirus*, and *Cegacovirus*) of the *Gammacoronavirus* genus. (**B**) Gammacoronaviruses of the *Igacovirus* subgenus, DuCoV2714 species. Colored font represents individual bird orders: black indicates *Anseriformes*, green indicates *Charadriiformes*, and red indicates other bird orders. The tree was constructed using MEGA 7 using the maximum likelihood method based on the T92 + G model and 1000 bootstrap replicates (bootstrap values shown on the tree).

One hundred and sixty-one viral sequences were submitted to GenBank, but in order to make the phylogenetic tree more readable, 72 were selected from this number, including 46 gamma- and 26 delta-coronaviruses. Our selection was based on the quality of the sequences obtained and their representativeness for given bird order/species. Of the 46 gammaCoVs analyzed in this study, 38 strains belonged to the *Igacovirus* subgenus, with 33 igacoviruses more related to the representative of the DuCoV2714 species. Only two strains clustered on the branch were occupied by two representatives of avian coronavirus AvCoV/AvCoV9203. Additionally, three strains along with seven others from different parts of the world formed a separate phylogenetic group. Interestingly, most of them came from pigeons, which is why they were called the “pigeon-like” group. Eight Polish strains were on the branches of the viruses of the *Brangacovirus* subgenus together with representative species of BcanCoVCB17 (Figure 1A).

Igacoviruses from the DuCoV2714 group were located on the two branches of the phylogenetic tree, but did not form clearly distinct groups supported by a high value of the bootstrap value (Figure 1B). Most of them were located in subgroup 1 together with the DuCoV2714 strain. Interestingly, the birds of *Anseriformes* predominated as hosts (11 mallards, 4 common teals, 3 mute swans, and 1 graylag goose). Only three igacoviruses from this group were identified in birds of different orders (one black-headed gull, one common gull, and one Eurasian coot). Subgroup 2 viruses were found mainly in birds of *Charadriiformes*—out of ten Polish strains, eight were identified in this order (six black-headed and two common gulls), and the remaining two strains were from mallards. Other birds hosting this subgroup include the *Charadriiformes* from Finland/arctic regions of Russia and the common snipe (*Scolopacidae*) from Australia. Among the two igacoviruses of the species AvCoV/AvCoV9203 identified, one was identified in graylag goose and one in pheasant (Figure 1A). A group that clearly differed from other igacoviruses was the “pigeon-like” group, which included strains identified only in pigeons across a large geographical area (various regions of China and Finland; Figure 1A). This group included three Polish strains—two identified in pigeons with 100% nucleotide homology and the third one in common starling, with a similarity of 97.3% to the others. Eight gammaCoVs identified in *Anseriformes* (four mute swans, three graylag goose, and one bean goose) were brangacoviruses (Figure 1A). Other *Brangacovirus* strains located in this phylogenetic branch have been identified in *Anseriformes* in Hong Kong, Finland, Arctic Russia, and Canada, but also in *Gruiformes* and *Pelecaniformes* in Africa (Madagascar). The nucleotide homology of the Polish strains was 96.2–100% with each other, and 95.2–96.4% with the subgenus BcanCoVCB17 strain.

The SH-aLRT values (SH > 0.9) within the branches formed by individual species were within the ranges, confirming their dissimilarity. A high value of SH = 0.99 was also obtained for the “pigeon-like” branch. In contrast, the SH value between the groups of the two DuCoV2714 subgroups was lower than 0.7.

### 3.3. Phylogenetic Analysis of DeltaCoV

**Figure 2:**
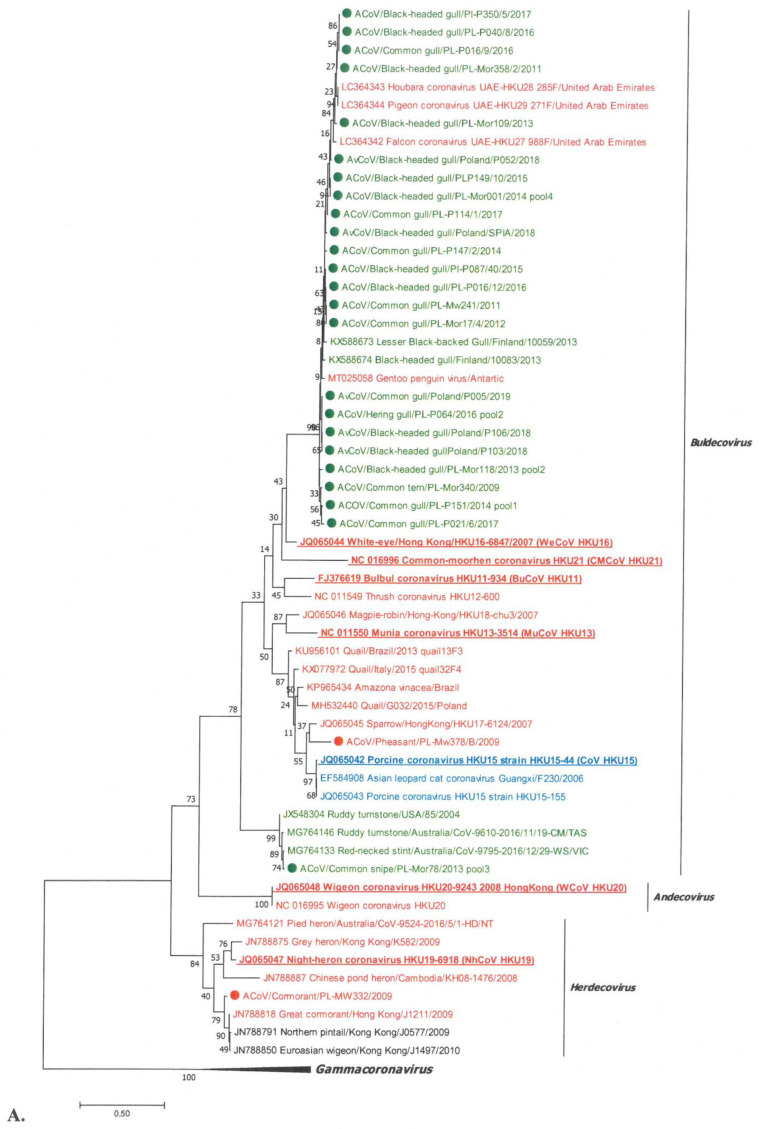

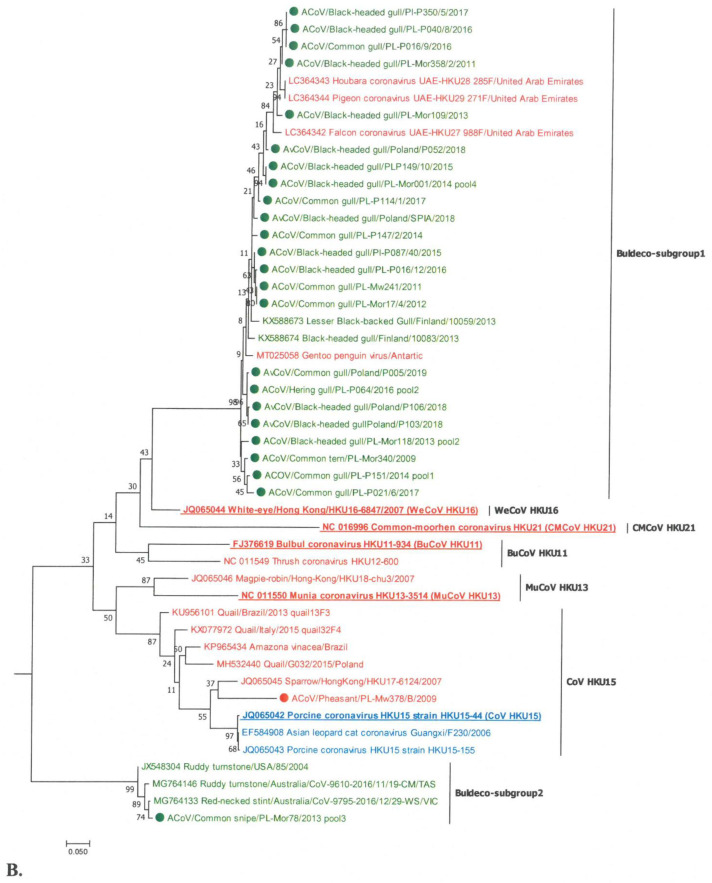
Phylogenetic analysis of deltacoronaviruses based on the replicase gene fragment. (**A**) The tree constructed for 58 deltacoronaviruses: 26 strains identified in wild birds in Poland (marked with a dot) and 32 from GenBank, including seven reference strains (written in bold and underlined) representing ratified species of three subgenera (five of *Buldecovirus*, and one for *Ardecovirus* and *Herdecovirus*) of the *Deltacoronavirus* genus. (**B**) Deltacoronaviruses of the *Brangacovirus* subgenus. Colored font for given bird orders: black indicates *Anseriformes*, indicates *Charadriiformes*, red indicates other bird orders, and blue indicates mammal species. The tree was constructed using MEGA 7 using the maximum likelihood method based on the GTR + G model and 1000 bootstrap replicates (bootstrap values shown on the tree).

Out of the 26 analysed deltaCoVs, one strain belonged to the lineage represented by the CoV HKU15 species of the *Buldecovirus* subgenus (Figure 2). In turn, the strain detected in the great cormorant was on a common branch within the *Herdecovirus* subgenus. The remaining 24 virus strains were in branches distinct from the others. Twenty-three deltaCoVs were located in the same lineage, with deltaCoVs detected in hubara bustards, pigeons, and falcons in the Middle East; black-headed gulls in Finland; and Gentoo penguin in Paradise Harbor, Antarctica. A subsequent strain identified in the common snipe was grouped in the branch occupied by CoV strains found in *Charadriiformes* in the USA and Australia (ruddy turnstone and red-necked stint). In the phylogenetic tree shown in Figure 2B, these two subgroups were designated as Buldeco-subgroup1 and Buldeco-subgroup2.

The calculated values of SH-aLRT of the branches formed by individual deltaCoV species were within the ranges, confirming their dissimilarity (SH > 0.9). High SH-aLRT values were also obtained for the branches occupied by deltaCoVs detected in Poland, and they were 0.98 and 1 for Buldeco-subgroup1 and Buldeco-subgroup2, respectively.

### 3.4. Annual Dynamics of Coronavirus Prevalence in Wild Birds

There was a seasonal difference in CoV detection in mallards and gulls, with a higher virus prevalence in August–November, then in December–February and in March–July, and the calculated differences were statistically significant (for gulls χ^2^ = 8.09, *p* = 0.01743; mallard ducks χ^2^ = 20.09, *p* = 0.000043). No statistically significant differences were observed in the mute swan population with regard to seasonality.

### 3.5. Results of Serology

Out of all 264 sera tested, antibodies against IB-like viruses were identified in 61 samples, representing 23.1% (Table 2). Surprisingly, the majority of the positive sera originated from white storks, which accounted for 64.8% positive results. Out of 87 sera collected from mallards, only 4 (4.6%) reacted positively in the ELISA test used. No antibodies against IBV were found in any of the tested mute swan sera.

## 4. Discussion

The large-scale study presented here revealed 4.15% prevalence of CoV in wild bird populations in Poland. Taking into account both the individual and pooled samples tested, among the 80 examined species belonging to 15 orders, CoVs were found in 16 of them, representing seven orders: six species of *Anseriformes* (mallard, common teal, tufted duck, mute swan, graylag, and bean goose); five species of *Charadriiformes* (common, black-headed and herring gull, common tern, and common snipe); and one bird species for *Passeriformes* (common starling), *Columbiformes* (pigeon), *Gruiformes* (Eurasian coot), *Suliformes* (great cormorant), and *Galliformes* (pheasant) (Table 1). Previously, CoV had been detected in 108 wild bird species belonging to 15 orders and 30 families [12]. They were most often found in water- and shore-birds of the orders *Anseriformes*, *Charadriiformes*, *Pelecaniformes*, *Suliformes*, and *Sphenciformes*, in synanthropic birds closely associated with humans and agriculture and urban areas (*Galliformes*, *Columbiformes*, and *Passeriformes*), and additionally in *Gruiformes*, *Accipitriformes*, *Strigiformes*, and *Falconiformes*. Our study confirms the presence of CoVs in the previously described species, and further demonstrates the virus in others not previously listed as CoV-positive (bean goose, common gull, common snipe, common tern, Eurasian coot, and common starling), which gives a total of 114 CoV-positive wild bird species. These birds, however, belong to orders/families previously confirmed as CoV-positive. The main reservoir of CoVs were *Anseriformes* and *Charadriiformes*, in which their prevalence was 3.51% and 5.59%, respectively. Similar studies conducted in other geographical regions showed various levels of CoV prevalence, from 1.6% in Northern England, 5.4% in Finland, 15% in Australia, and 18.7% in Sweden [17,18,19,20]. Such a large variation may result from the use of different molecular methods, different numbers of samples tested, the species included in the study, and the geographical region where the samples were collected [12].

GammaCoVs were detected in six orders of birds with a prevalence of 3.5%. Only gammaCoVs were detected in *Anseriformes* (3.51%), while their presence was also found in *Charadriiformes* with a similar frequency (3.14%). In addition, these viruses were also detected in *Columbiformes*, *Galliformes*, *Gruiformes*, and *Passeriformes*. In contrast, the prevalence of deltacoronaviruses was lower and amounted to 0.65%. The presence of these viruses was detected in birds belonging to three orders, *Charadriiformes*, *Galliformes*, and *Suliformes*, which may indicate a narrower host range than for gammaCoV. Deltacoronaviruses were most frequently detected in gulls, which appeared to be a common host for both gamma- and delta-CoV. This phenomenon is similar to what has been observed with regard to avian influenza virus, as a number of subtypes of this virus were detected in *Laridae* family, including the most important, H5, H7, and H9. This may be due to the specificity of this bird family. Shorebirds are found all over the world, occupying diverse ecologic niches. They also differ in their ability to migrate—some migrate between continents, and others are sedentary. Furthermore, shorebirds are species that are ecologically associated with humans and domestic animals, ranging from frequent direct contact resulting from sharing the same environment, to very occasional contact. The diet of *Laridae* is also a factor contributing to the infection of various viruses. They are generally omnivores, although they are most likely to consume invertebrates and fish—some species eat sick or dead animals, thus facilitating the direct transmission of various pathogens. Moreover, *Laridae* breed in high-density colonies, where contact between infected individuals can easily occur [21].

Within *Anseriformes*, gammaCoVs were most often identified in mallards and mute swans, with prevalence of about 4% and 2%, respectively. The occurrence of gammaCoV in mallards has been previously reported several times in Sweden, Norway, Finland, Poland, and the USA [13,19,20,22,23,24,25]. In total, over 5300 mallards were tested, detecting gammaCoVs in almost 400 birds and giving an overall prevalence of about 7.4% (range from 0.6% to over 21%) [12]. Interestingly, our previous results on IBV-like CoVs identified a similar percentage of positive mallards (12 positive/292 tested, 4.1%). The results of the studies on the presence of gammaCoV in mute swans are also quite interesting. So far, their presence has only been identified in Poland. In a previous study conducted between 2008 and 2011 on 161 examined individuals of this species, gammaCoVs were identified in two species (1.2%) [13]. Within *Charadriiformes*, gammaCoVs were identified in *Laridae*, in 5.6% and 3.1% of the examined common and black-headed gulls, respectively, and these results confirm previous observations [19,23,26,27,28]. Interestingly, we also identified a high number of pigeons (45.0%) infected with gammaCoV, although the number of samples tested was relatively small (*n* = 20). The frequent occurrence of gammaCoVs in pigeons was previously observed by Zhuang et al. (2020) in China, where the prevalence was over 23% [29].

DeltaCoVs were identified in five species of the *Charadriiformes*, the great cormorant of the *Suliformes* and in pheasant of the *Galliformes*. While results reporting the identification of deltaCoVs in *Charadriiformes*, including *Laridae*, and also in *Suliformes* were previously reported, the presence of this virus in pheasants is worth noting [18,19,26,28,30]. There were many studies reporting the susceptibility of pheasants to infections caused by IBV-like CoV [31,32,33]. To date, the only *Galliformes* representative infected with deltaCoV was quail. Information on the presence of deltaCoV in this bird species comes from studies of free-living birds conducted in the United Arab Emirates, and from cases in farmed quail flocks in Brazil and Poland [11,34,35,36].

A phylogenetic analysis of the gammaCoVs identified in Poland showed that they belong to two subgenera *Igacovirus* and *Brangacovirus*. Most igacoviruses formed a common branch of the phylogenetic tree with a representative of the DuCoV2714 species. Interestingly, the DuCoV2714 strains formed two branches, the subgroup1 clustered strains from *Anseriformes* and the subgroup2 strains from *Charadriiformes*; however, these subgroups were not homogeneous, as each included single strains from other bird orders. A similar relationship was reported previously. An analysis of a 464 nt fragment of the RdRp gene identified in wild birds in Finland showed a separate grouping of strains found in ducks from those detected in different species of gulls [19]. In addition, Chamings et al. (2018), on the basis of a phylogenetic analysis of an RdRp fragment of much smaller size (277 nt), showed clustering of gammaCoV from *Anseriformes* and *Charadriiformes* on separate branches [18]. However, such a relationship is not the principle among gammaCoVs, and published results have repeatedly shown the presence of strains from birds belonging to different taxonomic orders within a single branch of phylogenetic tree [13,19,23,37]. Thus, it seems that some igacoviruses are species-specific and others can also spread between different bird species. It is possible that there may be a relationship that has been observed with AIV. Studies on the correlation between AIV prevalence and species, geographic location, and sampling time showed weak support for species dependence, while evidence for phylogenetic clustering in location and time was found [38,39]. Among the analyzed strains of the *Igacovirus* subgenus, only two, identified in greylag goose and common pheasant, were in the AvCoV/AvCoV9203 group. This group also included two Polish strains previously identified in greylag goose and mallard, as well as igacoviruses identified in wild birds (quail, egret, teal, or house sparrow) in Africa [13,37,40]. Most likely, the presence of these viruses in wild birds is the result of their spill over from poultry [40,41]. Interestingly, AvCoV/AvCoV9203 viruses have been identified in numerous African wild birds (Egypt, Madagascar). It is possible that this is due to the way poultry is reared on this continent (most probably semi-open). The third group of igacoviruses was formed by the strains identified in pigeons (*Columbiformes*) and common starling (*Passeriformes*), designated as the “pigeon-like” group. This group also included pigeon coronavirus strains from Finland and China [19,29]. Recently, Chinese researchers found that pigeon CoV strains formed a distinct phylogenetic lineage compared to other poultry (chicken and duck) coronaviruses, and even concluded that pigeon coronavirus is a distinct species within the Igacovirus subgenus [29]. Our results confirm the high specificity of such coronaviruses for pigeons. The presence of a similar virus found in the common starling could be a consequence of contacts with pigeons. The pigeon is a known species of synanthropic bird, often living in environments close to humans. Starlings have also, in response to progressive urbanization, adapted to life in the vicinity of humans, and they are frequent residents of gardens, parks, and farms, and can sometimes even be found in the centers of large cities. Of the 46 gammaCoV strains analyzed, eight were classified into the *Brangacovirus* subgenus. These brangacoviruses were identified in *Anseriformes*, and grouped together with other viruses mainly from birds of this order identified in areas of Finland, Sweden, and China, described at the time as gammaCoVs belonging to cluster B [19,23,42].

A phylogenetic analysis of 26 detected deltacoronaviruses revealed that most of them belonged to the *Buldecovirus* subgenus and only one belonged to *Herdecovirus*. No virus of the *Andecovirus* subgenus was detected. The indicated viruses of the *Buldecovirus* subgenus are characterized by a high genetic variation. Strains from gulls formed the Buldeco-subgroup1, with viruses detected in gulls from Finland, but also in other bird species such as hubara bustard, pigeon, and falcon from the Middle East and Gentoo penguin from the Antarctic [11,12,30,43]. However, these viruses are not represented by any ratified virus species, although such a need has been previously indicated [11,12]. The fact that of the 26 deltacoronaviruses analyzed, as many as 23 from *Laridae* belonged to the same *Buldecovirus* subgroup1, suggests the species specificity of this group. Although viruses from birds of different families (bustards, pigeons, falcons, and penguins) were also included in this subgroup, their occurrence appears to be sporadic and may result from sharing the same habitat with gulls (e.g., penguins) or their eating habits (the presence of similar viruses in falcon, hubara, and pigeon caught in the same area was interpreted as a consequence of the food chain) [11]. Another interesting buldecovirus strain from *Charadriiformes* has been identified in common snipe of the *Scolopacidae*. This strain formed a separate Buldeco-subgroup2 together with deltaCoVs found in other *Scolopacidae* species, i.e., in ruddy turnstone from the USA and Australia, and red-necked stint from Australia [18,44]. As before, the distinctiveness of this group was supported by the high value of the calculated SH-aLRT coefficient, and the lack of a representative of the group as a ratified species was also noted. However, while the viruses clustered in the Buldeco-subgroup1 mainly originated from the birds of *Laridae*, as well as from other orders, the Buldeco-subgroup2 seemed to be highly species specific only for birds of *Scolopacidae*. Only one buldecovirus identified in the pheasant of the *Galliformes* was placed in the subgroup represented by the species ratified by ICTV—porcine coronavirus HKU15. This subgroup also included other mammalian deltaCoV, from pigs and Asian leopard cat, as well as birds, detected in species such as sparrow, quail, and red amazon [9,45,46,47]. The only deltacoronavirus of the *Herdecovirus* subgenus was identified in the cormorant. Interestingly, viruses of this subgenus seem to be species specific, as most were detected in birds of *Pelecaniformes*, as they were identified in the herons in Australia, and in herons and cormorants in the Mai Pohes nature reserve in Hong Kong [18,42]. The presence of herdecoviruses has also been identified in *Anseriformes*, northern pintail, and Eurasian wigeon, but rather sporadically [42].

This study revealed the formation of phylogenetic groups of “species-specific” viruses with a high homology for both gamma- and delta-coronaviruses from various geographical areas, sometimes very distant, e.g., cormorant herdecoviruses from Poland and Hong Kong or pigeon igacoviruses from Poland and China. This is likely due to bird behavior—seasonal migrations associated with wintering/breeding. Although some birds are sedentary (mallards and pigeons), contact with migratory birds could not be excluded. In turn, some birds migrate short distances, but have contact with others that migrate further—virus transmission may be a “relay race”. It should be emphasized that the territory of Poland is one of the most important wintering and breeding grounds and bird migration routes.

A commercial ELISA was used to test the level of antibodies against IB-like viruses in the sera of wild birds. Out of the 264 tested sera, positive results were recorded for 61 samples, which accounted for 23.1%. Especially surprising was the high percentage of positive sera (64.8%) from white storks. However, it should be added that these sera came from storks staying in Rehabilitation Centers, where the birds were fed mainly with poultry meat or even few-day-old chicks. As is commonly known, chickens are repeatedly vaccinated with live attenuated vaccines, which may explain the presence of antibodies (a consequence of oral contact with the virus). The serological tests also showed that only 4.6% of mallard sera were positive, which may be the result of infection with an IB-like virus. In the present study, infection with igacovirus AVCoV/AvCoV9203 was found only in greylag goose and pheasant, but in our previous studies, such infections were also identified in mallards [13]. A similar study in wild birds conducted in Nigeria in 2017 showed 40.5% seroprevalence [48]. The highest number of positive sera was found in herons, Canada goose, and mallards, with a prevalence of 70.4%, 64.3%, and 63.6%, respectively. In all of these species, the presence of gammaCoV has been molecularly detected, which may explain the presence of antibodies.

The obtained results also revealed that coronavirus prevalence in the wild bird population in Poland depends on the season. Statistically significant differences were found in CoV prevalence in mallards and gulls; the highest rates of CoV infections in both groups were found in late summer/early fall. Similar infection dynamics were demonstrated for avian influenza virus and coronavirus in mallards in Sweden [25]. The number of juveniles susceptible to all infections is the highest in autumn, and the birds are intensively preparing for migration, often clustering into large groups, which favors the transmission of pathogens between individuals.

## 5. Conclusions

In conclusion, the results obtained indicate the presence of both gamma- and delta-coronaviruses in the wild bird population in Poland, although these infections are not common, and the prevalence of deltacoronaviruses is much lower than gammacoronaviruses. Furthermore, the analysis showed that these viruses are genetically diverse. We also reveal gulls as hosts for both gamma- and delta-coronaviruses, with both viruses clearly exhibiting a “species-dependent” grouping. This phenomenon is similar to what has been observed with regard to avian influenza virus, as a number of virus subtypes were detected in the *Laridae* family, including the most important H5, H7, and H9. The results obtained also indicate a relatively low prevalence of IB-like gammacoronaviruses in wild bird populations. It seems important to obtain whole-genome sequences of the detected viruses, including those from *Laridae*, and such studies are currently underway. Moreover, taking into account the high variability of coronaviruses and their ability to break the species barrier, it seems justified to continue such studies in the future.

## Figures and Tables

**Table 1:** Birds screened in this surveillance and an overview of the prevalence of coronaviruses in the studied samples.

Avian Order/Family	Common Name	Species	Individuals Studied	Positive (Gamma/Delta)	Pooled Studied	Positive (Gamma/Delta)
*Anseriformes*/*Anatidae*	Mallard	*Anas platyrhynchos*	1566	63 (63/0)	65	5 (5/0)
	Common teal	*Anas crecca*	104	12 (12/0)	2	1 (1/0)
	Garganey	*Anas querquedula*	2	0	1	0
	Gadwall	*Anas strepera*	4	0	-	-
	Tufted duck	*Aythyda fuligula*	2	1 (1/0)	-	-
	Northern pintail	*Anas acuta*	1	0	-	-
	Carolina duck	*Aix sponsa*	1	0	-	-
	Longnatailed duck	*Clangula hyemalis*	1	0	-	-
	Mandaryn duck	*Aix galericulata*	1	0	-	-
	Common pochard	*Aythya ferina*	2	0	-	-
	Velvet scoter	*Melanitta fusca*	4	0	-	-
	Eurasian wigeon	*Mareca penelope*	1	0	-	-
	Mute swan	*Cygnus olor*	1119	23 (23/0)	85	6 (6/0)
	Whooper swan	*Cygnus cygnus*	5	0	3	0
	Tundra swan	*Cygnus columbianus*	*-*	-	1	0
	Greylag goose	*Anser anser*	2	0	100	7 (7/0)
	Bean goose	*Anser fabalis*	3	0	27	2 (2/0)
	White-fronted goose	*Anser albifrons*	4	0	2	0
	Egyptian goose	*Alopochen aegyptiaca*	-	-	1	0
	Barnacle goose	*Branta leucopsis*	-	-	2	0
	Canada goose	*Branta canadensis*	-	-	1	0
	Brent goose	*Branta bernicla*	-	-	1	0
*Charadriiformes*/*Laridae*	Common gull	*Larus canus*	142	18 (8/10)	25	3 (3/0)
	Black-headed gull	*Larus ridibundus*	773	35 (24/11)	30	0
	European herring gull	*Larus argentatus*	82	3 (0/3)	6	1 (1/0)
	Great black-backed gull	*Larus marinus*	4	0	-	-
	Mediterranean gull	*Larus melanocephalus*	2	0	-	-
	Caspian gull	*Larus cachinnans*	4	0	-	-
*Charadriiformes*/*Sternidae*	Common tern	*Sterna hirundo*	3	0	5	1 (0/1)
*Charadriiformes*/*Charadriidae*	Northern lapwing	*Vanellus vanellus*	-	-	1	0
*Charadriiformes*/*Scolopacidae*	Wood sandpiper	*Tringa glareola*	1	0	-	-
	Ruff	*Calidris pugnax*	1	0	-	-
	Common snipe	*Gallinago gallinago*	5	1 (0/1)	-	-
	Redshank	*Tringa totanus*	1	0	-	-
*Charadriiformes*/*Alcidae*	Common guillemot	*Uria aalge*	1	0	-	-
	Lesser auk	*Alca torda*	1	0	-	-
*Galliformes*/*Phasianidae*	Common pheasant	*Phasianus colchicus*	2	1 (0/1)	6	1 (1/0)
	Common quail	*Coturnix coturnix*	-	-	1	0
	Grey partridge	*Perdix perdix*	3	0	4	0
*Gruiformes*/*Rallidae*	Eurasian coot	*Fulica atra*	29	0	5	1 (1/0)
	Common moorhen	*Gallinula chloropus*	2	0	-	-
*Gruiformes*/*Gruidae*	Common crane	*Grus grus*	1	0	6	0
*Suliformes*/*Phalacrocoracidae*	Great cormorant	*Phalacrocorax carbo*	24	0	2	1 (0/1)
*Podicipediformes*/*Podicipedidae*	Great crested grebe	*Podiceps cristatus*	2	0	2	0
*Columbiformes*/*Columbidae*	Pigeon	*Columba livia*	19	9 (9/0)	2	0
	Common wood pigeon	*Columba palumbus*	1	0	-	-
	European turtle dove	*Streptopelia turtur*	-	-	1	0
	Eurasian collared dove	*Streptopelia decaocto*	2	0	-	-
*Passeriformes*/*Corvidae*	Carrion crow	*Corvus corone*	2	0	1	0
	Rook	*Corvus frugilegus*	5	0	2	0
	Eurasian jay	*Garrulus glandarius*	1	0	-	-
	Eurasian magpie	*Pica pica*	1	0	-	-
*Passeriformes*/*Passeridae*	House sparrow	*Passer domesticus*	-	-	1	0
	Eurasian tree sparrow	*Passer montanus*	2	0	-	-
*Passeriformes*/*Turdidae*	Common blackbird	*Turdus merula*	2	0	-	-
	Fieldfare	*Turdus pilaris*	1	0	-	-
*Passeriformes*/*Sturnidae*	Common starling	*Sturnus vulgaris*	-	-	2	1 (1/0)
*Passeriformes*/*Certhiidae*	Shortnatoed treecreeper	*Certhia brachydactyla*	1	0	-	-
*Passeriformes*/*Hirundinidae*	Barn swallow	*Hirundo rustica*	1	0	1	0
*Passeriformes*/*Fringillidae*	European greenfinch	*Chloris chloris*	3	0	-	-
	Common chaffinch	*Fringilla coelebs*	1	0	-	-
	Brambling	*Fringilla montifringilla*	1	0	-	-
*Passeriformes*/*Paridae*	Eurasian blue tit	*Cyanistes caeruleus*	1	0	-	-
	Great tit	*Parus major*	4	0	-	-
*Accipitriformes*/*Accipitridae*	Northern goshawk	*Accipiter gentilis*	3	0	2	0
	Common buzzard	*Buteo buteo*	2	0	-	-
	White-tailed eagle	*Haliaeetus albicilla*	3	0	-	-
	Western marsh harrier	*Circus aeruginosus*	1	0	-	-
	Lesser spotted eagle	*Clanga pomarina*	1	0	-	-
	Eurasian sparrowhawk	*Accipiter nisus*	1	0	-	-
*Falconiformes*/*Falconidae*	Peregrine falcon	*Falco peregrinus*	2	0	1	0
	Common kestrel	*Falco tinnunculus*	2	0	-	-
*Pelecaniformes*/*Arteidae*	Great egret	*Ardea alba*	-	-	2	0
	Grey heron	*Ardea cinerea*	4	0	1	0
*Ciconiiformes*/*Ciconiidae*	White stork	*Ciconia ciconia*	21	0	-	-
*Gaviiformes*/*Gaviidae*	Red-throated loon	Gavia stellata	1	0	-	-
*Strigiformes*/*Strigidae*	Brown owl	*Strix aluco*	1	0	-	-
	Little owl	*Athene noctua*	1	0	-	-
	Long-eared owl	*Asio otus*	1	0	-	-
*Apodiformes*/*Apodidae*	Common swift	*Apus apus*	1	0	-	-
**Total**			**3998**	**166 (140/26)**	**400**	**30 (28/2)**

**Table 2:** Results of serological test (antibodies against IBV) in the tested wild bird serum.

Avian Order/Family	Common Name	Species	Sera Studied	No. of Positives	%
*Anseriformes*/*Anatidae*	mallard	*Anas platyrhynchos*	87	4	4.6
	mute swan	*Cygnus olor*	89	0	-
*Ciconiiformes*/*Ciconiidae*	white stork	*Ciconia ciconia*	88	57	64.8
**Total:**			**264**	**61**	**23.1**

## Data Availability

The genome sequences generated in this study were submitted to the GenBank database (https://www.ncbi.nlm.nih.gov/genbank/) (accessed on 6 October 2019). Under accession numbers MK617357-MK617517.
